# Imaging Findings of Human Papillomavirus-Positive and Human Papillomavirus-Negative Oropharyngeal Squamous Cell Carcinoma Associated with Recurrence

**DOI:** 10.3390/jcm14031027

**Published:** 2025-02-06

**Authors:** Taketo Suto, Masaya Kawaguchi, Hiroki Kato, Hirofumi Shibata, Takenori Ogawa, Tomohiro Ando, Yoshifumi Noda, Fuminori Hyodo, Masayuki Matsuo

**Affiliations:** 1 Department of Radiology, Gifu University, 1-1 Yanagido, Gifu 501-1194, Japan; suto.taketo.k1@f.gifu-u.ac.jp (T.S.); kato.hiroki.w4@f.gifu-u.ac.jp (H.K.); ando.tomohiro.y6@f.gifu-u.ac.jp (T.A.); noda.yoshifumi.g9@f.gifu-u.ac.jp (Y.N.); matsuo.masayuki.e0@f.gifu-u.ac.jp (M.M.); 2 Department of Otolaryngology, Gifu University, Gifu 501-1194, Japan; shibata.hirofumi.b3@f.gifu-u.ac.jp (H.S.); ogawa.takenori.d2@f.gifu-u.ac.jp (T.O.); 3 Center for One Medicine Innovative Translational Research (COMIT), Institute for Advanced Study, Gifu University, Gifu 501-1194, Japan; hyodo.fuminori.i4@f.gifu-u.ac.jp

**Keywords:** human papillomavirus 16, oropharyngeal squamous cell carcinoma, prognosis, MRI, CT

## Abstract

**Objectives:** This study aimed to compare the imaging findings associated with the recurrence of HPV-positive and HPV-negative oropharyngeal squamous cell carcinoma (OPSCC). **Methods:** In total, 68 patients (51 men; mean age, 64.4 years; age range, 41–86 years; 48 HPV-positive patients and 20 HPV-negative patients) with histopathologically proven OPSCC who underwent CT, MRI, and 18F-FDG-PET/CT before treatment between October 2014 and July 2022 were enrolled in this study. The imaging findings were retrospectively evaluated and statistically compared. **Results:** HPV-positive OPSCC had a significantly lower recurrence rate compared with that of HPV-negative OPSCC (*p* < 0.01). Among HPV-positive OPSCCs, patients with recurrence were considerably older than those without recurrence (*p* < 0.05); however, the T and N categories did not differ between the two groups. Meanwhile, among HPV-negative OPSCCs, the T and N categories were associated with recurrence (*p* < 0.05). Furthermore, the attenuation on contrast-enhanced CT (*p* < 0.05) and signal intensity on contrast-enhanced T1-weighted images (*p* < 0.05) of nodal metastases were significantly lower in recurrence patients compared to those in nonrecurrence patients. Cystic change in nodal metastases in HPV-positive and HPV-negative OPSCCs were similar in patients with and without recurrence. **Conclusions:** The T and N categories were associated with recurrence in HPV-negative OPSCC but not in HPV-positive OPSCC. Prognostic factors differed significantly between HPV-positive and HPV-negative OPSCC.

## 1. Introduction

Oropharyngeal squamous cell carcinoma (OPSCC) comprises cancers of the tonsils, base of the tongue, soft palate, and uvula. OPSCC is one of the most rapidly growing subtypes of head and neck cancers and has historically been linked to alcohol and tobacco consumption. Recently, the role of human papillomavirus (HPV) in carcinogenicity was confirmed, and HPV infection has become widely accepted as a cause of OPSCC [1,2,3].

OPSCC is classified into two disease entities with distinct clinical and molecular characteristics based on HPV involvement. HPV-positive OPSCC develops at a younger age when compared with HPV-negative OPSCC, regardless of smoking or drinking [4]. Because significant differences in clinical prognosis have been discovered between HPV-positive and HPV-negative OPSCC, making it challenging to accurately predict prognosis within the same category, the American Joint Committee on Cancer’s eighth edition of the TNM classification system was revised to include a new classification for HPV-positive and HPV-negative OPSCC [5].

Several previous studies have reported imaging findings of HPV status in OPSCC and prognostic factors for overall or HPV-positive OPSCC alone. Cystic lymph node metastasis was more common in HPV-positive OPSCC than in HPV-negative OPSCC [6]; therefore, OPSCC with cystic nodal metastasis had a favorable prognosis. Imaging predictors of poor prognosis in HPV-positive OPSCC included low-neck or retropharyngeal lymphadenopathy [7] and extranodal extension (ENE) [8,9,10]. Furthermore, CT-based radiomics could potentially aid prognostication for patients with OPSCC [11]. However, to the best of our knowledge, few studies have examined the prognostic value of imaging findings for HPV-negative OPSCC alone or HPV-positive and HPV-negative OPSCC separately. This study aimed to identify CT, MRI, and 18F-fluorodeoxyglucose (FDG)-PET/CT findings of HPV status in OPSCC to predict recurrence.

## 2. Materials and Methods

### 2.1. Patients

The Institutional Review Board of our hospital (Gifu University, 2020-101) approved this study, which follows the guidelines of the Health Insurance Portability and Accountability Act of 1996 and the Helsinki Declaration. Informed consent was waived because of the study’s retrospective nature. From October 2014 to July 2022, we searched our hospital’s electronic medical record system for OPSCC that had been pathologically diagnosed following CT, MRI, and 18F-FDG-PET/CT procedures. HPV status was determined by p16-INK4a immunohistochemistry staining. Positive p16 expression was defined as strong and diffuse nuclear and cytoplasmic staining in at least 70% of tumor specimens [12]. Exclusion criteria were as follows: (1) patients without CT, MRI, or 18F-FDG-PET/CT and (2) undetermined HPV status. Thus, the study identified and included 68 patients with OPSCC (51 men, 17 women; mean age, 64.4 years; age range, 41–86 years; 48 HPV-positive patients and 20 HPV-negative patients). Furthermore, we searched electronic medical records for medical and preference histories at the first visit, the therapeutic course, and post-treatment recurrence.

### 2.2. Imaging Technique

All patients had their MRI performed on 1.5T scanners (Intera Achieva 1.5T Pulsar or Ingenia Provida 1.5T; Philips Healthcare, Best, the Netherlands) or a 3T scanner (Ingenia 3.0T CX; Philips Healthcare, Best, the Netherlands). All transverse MRI images were obtained with a 3–4 mm section thickness and a 1 mm intersection gap. All patients had undergone T1-weighted fast spin-echo (TR/TE, 620–780/9–18 ms; field of view, 20 × 20 cm), T2-weighted spin-echo (TR/TE, 3200–5710/90 ms; field of view, 20 × 20 cm), and diffusion-weighted short-tau inversion recovery single-shot spin-echo echo-planar (TR/TE/TI, 4940–18600/65–72/170–240 ms; field of view, 24 × 24–40 × 40 cm; b value, 0 and 1000 s/mm^2^) images. Fat-suppressed gadolinium-enhanced T1-weighted spin-echo (TR/TE, 630–680/9–19 ms; field of view, 20 × 20 cm) images were obtained from all patients following intravenous injection of 0.1 mmol/kg gadopentetate dimeglumine (Magnevist, Bayer HealthCare, Leverkusen, Germany) or gadobutrol (Gadavist, Bayer HealthCare, Leverkusen, Germany).

CT imaging was performed on all patients using a 16-slice scanner (LightSpeed 16; GE Healthcare, Milwaukee, WI, USA), a 64-slice scanner (Brilliance CT 64 or Discovery CT750HD; GE Healthcare, Milwaukee, WI, USA), or a 512-slice scanner (Revolution CT; GE Healthcare). Axial unenhanced and enhanced CT images were obtained from all patients and reconstructed at a section thickness of 2.5 mm without overlap. Single-phase contrast-enhanced CT imaging was initiated 45 s after initiating an intravenous bolus injection of 100 mL of nonionic iodine contrast material (Optiray 240 [240 mg of iodine per ml]; Mallinckrodt Inc., Hazelwood, MO, USA) at an injection rate of 2 mL/s.

For all patients, whole-body PET/CT (Biograph Sensation 16; Siemens Medical Solutions or Discovery MI; GE Healthcare, Milwaukee, WI, USA) from the skull to the mid-thigh was performed. The patients received an intravenous injection of 18F-FDG following a minimum of 4 h of fasting. Approximately 60 min after 18F-FDG injection, CT and subsequent whole-body PET were performed. Transverse images were reconstructed using a 2 mm section thickness with no overlap. A 256 × 256 imaging matrix with a 50 × 50 cm field of view was used to obtain axial PET images.

### 2.3. Imaging Assessment

Two radiologists with 24 and 10 years of experience in head and neck imaging examined all images. The reviewers were blinded to all clinical information. Any disagreement among the reviewers was resolved via discussion to reach a consensus.

The reviewers qualitatively evaluated the primary lesions based on the margin (well-defined or ill-defined), necrosis (presence or absence), marginal invasion (presence or absence), and signal intensity relative to the cervical cord on T1-weighted, T2-weighted, contrast-enhanced T1-weighted, and diffusion-weighted images. Marginal invasion was defined according to the presence of moderately (T4a) or very advanced (T4b) local tumors. Subsequently, the reviewers assessed lymph node metastasis in terms of ENE (presence or absence), unenhanced area (presence or absence), and signal intensity relative to the cervical cord on T1-weighted, T2-weighted, contrast-enhanced T1-weighted, and diffusion-weighted images. Cervical lymph nodes that had a maximum diameter of 10 mm or more, contained central necrosis, or had indistinct margins were considered positive for metastasis. ENE was defined by poorly defined margins, thickening or enhancement of the nodal rim, irregularity of the nodal capsule, or evidence of infiltration into adjacent fat or other soft tissue [10,13]. The unenhanced area was categorized as follows: (1) pure cystic, a lesion with a cyst wall < 2 mm without any solid component; (2) pure cystic with a mural nodule, a pure cystic lesion with a mural nodule; and (3) others, a lesion with a solid component excluding the mural nodule and/or a cyst wall > 2 mm.

The attenuation (HU) on unenhanced and contrast-enhanced CT images and the signal intensity on T1-weighted, T2-weighted, contrast-enhanced T1-weighted, and diffusion-weighted images were measured for quantitative evaluation of the solid component of the primary lesions and lymph node metastases. The signal intensity of the primary lesion and lymph node metastasis excluding the necrotic area and that of the cervical cord at the same level were measured, and the signal intensity ratio (SIR) of the solid component to the cervical cord was computed. The apparent diffusion coefficient (ADC) value of the solid component was measured on ADC maps by placing regions of interest as broadly as possible over the lesion, excluding the necrotic area with reference to T2-weighted and contrast-enhanced T1-weighted images. The maximum standardized uptake value (SUVmax) of the lesion, defined as the highest voxel value among all tumor voxels, was measured.

### 2.4. Statistical Analysis

All statistical analyses were performed with EZR (Saitama Medical Center, Jichi Medical University, Saitama, Japan), which is a graphical user interface for R version 4.0 (The R Foundation for Statistical Computing, Vienna, Austria) [14]. The quantitative outcomes of OPSCC patients with and without recurrence were compared using the Mann–Whitney U test. The qualitative outcomes of patients with OPSCC with and without recurrence were compared using Fisher’s exact test. The statistical power between the 48 HPV-positive patients and 20 HPV-negative patients was 0.79. We set a significant level (α) at 0.05. If the *p* value falls below this threshold, the results are considered statistically significant. The interobserver variability of qualitative assessments was investigated using κ statistics.

## 3. Results

### 3.1. Patient Characteristics

**Figure 1:**
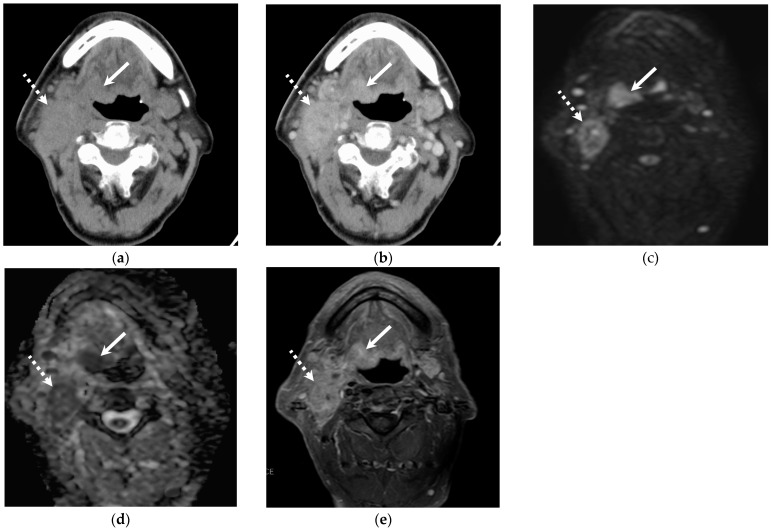
Representative images of an 86-year-old male with HPV-positive OPSCC in the base of the tongue. Local recurrence and pulmonary metastasis occurred. (**a**) Unenhanced CT shows soft tissue mass in the right base of the tongue (primary lesion, arrow) and right upper jugular lymph node metastasis (dotted arrow). CT attenuation of the nodal metastasis shows +42 HU. (**b**) Contrast-enhanced CT shows enhanced primary lesion (arrow) and nodal metastasis (dotted arrow). (**c**,**d**) Diffusion-weighted imaging and ADC map show high signal intensity and low ADC value in the primary lesion (arrow) and nodal metastasis (dotted arrow). (**e**) Fat-suppressed contrast-enhanced T1-weighted image shows primary lesion (arrow) and nodal metastasis (dotted arrow). The SIR of solid component is 2.15.

**Figure 2:**
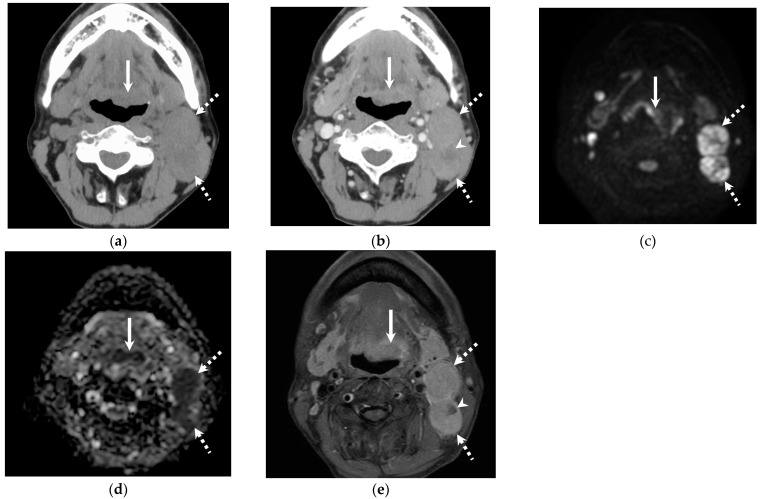
Representative images of a 58-year-old male with HPV-negative OPSCC in the base of the tongue. Local recurrence and pulmonary metastasis occurred. (**a**) Unenhanced CT shows mass in the base of the tongue (primary lesion, arrow) and multiple left upper jugular lymph node metastases (dotted arrow). CT attenuation of the primary lesion shows +44 HU. (**b**) Contrast-enhanced CT shows primary lesion and nodal metastasis. CT attenuation of nodal metastasis demonstrated + 75 HU. (**c**,**d**) Diffusion-weighted imaging and ADC map show high signal intensity and low ADC value. ADC value of the nodal metastasis was 0.715 × 10^−3^mm^2^/s. (**e**) Fat-suppressed contrast-enhanced T1-weighted image shows primary lesion (arrow) and multiple nodal metastases (dotted arrow) with necrosis (arrowhead). The SIR of solid component of the lymph node is 1.80.

Table 1 shows patient characteristics. The recurrence rate of OPSCC in HPV-positive patients was significantly lower than in HPV-negative patients (15% vs. 50%; *p* < 0.01). Age was considerably higher in HPV-positive OPSCC patients with recurrence than in those without recurrence (73 years vs. 60 years, *p* < 0.05); however, there was no significant difference in the T (*p* = 0.52) and N stages (*p* > 0.99) based on the eighth edition of the staging system between patients with and without recurrence. Meanwhile, among HPV-negative OPSCC patients, patients with recurrence had higher T (*p* < 0.05) and N stages (*p* < 0.05) than those without recurrence (Figure 1 and Figure 2).

### 3.2. Quantitative and Qualitative Imaging Findings of the Primary Lesion

Table 2 summarizes the quantitative and qualitative imaging findings of the primary lesion. SIR on diffusion-weighted (1.66 vs. 1.32; *p* < 0.001) and contrast-enhanced T1-weighted images (2.05 vs. 1.82; *p* = 0.024) was significantly higher in HPV-positive OPSCC patients with recurrence than in those without recurrence; however, there was no significant difference in ADC value (*p* = 0.98) or attenuation on contrast-enhanced CT images (*p* = 0.88). In HPV-negative OPSCC patients, attenuation on contrast-enhanced CT images (42.1 HU vs. 49.4 HU; *p* < 0.01) was significantly lower in patients with recurrence than in those without recurrence; however, there was no significant difference in SIR on contrast-enhanced T1-weighted images (*p* = 0.91). There was no significant difference in qualitative imaging findings between the primary lesions.

### 3.3. Quantitative Imaging Findings of Cervical Lymph Node Metastasis

Table 3 shows the quantitative imaging findings of cervical lymph node metastasis. Lymph node metastasis was observed in 57 (84%) of the 68 patients with OPSCC, and 153 nodal metastases were confirmed. Attenuation on unenhanced CT images of the solid component within HPV-positive nodal metastases (44.1 HU vs. 47.1 HU; *p* = 0.010) was significantly lower in patients with recurrence in comparison to those without. In HPV-negative nodal metastases, attenuation on contrast-enhanced CT (79.4 HU vs. 87.9HU; *p* < 0.05) and SIR on contrast-enhanced T1-weighted images (1.79 vs. 2.41; *p* < 0.05) of the solid component were significantly lower in patients with recurrence than in those without. In HPV-negative nodal metastases, the ADC value was marginally lower in patients with recurrence than in those without (0.83 × 10^−3^ mm^2^/s vs. 1.13 × 10^−3^ mm^2^/s; *p* = 0.058), and SUVmax was marginally higher in patients with recurrence (7.86 vs. 4.31; *p* = 0.070) (Figure 1 and Figure 2).

### 3.4. Qualitative Imaging Findings of Cervical Lymph Node Metastasis

Table 4 shows the qualitative imaging findings of cervical lymph node metastasis. In HPV-positive nodal metastasis, there was no significant difference in findings between patients with and without recurrence. In HPV-negative nodal metastasis, the frequency of ENE on CT (4% vs. 44%; *p* < 0.05) and MRI (4% vs. 44%; *p* < 0.05) images was significantly lower in patients with recurrence than in those without. Regardless of HPV status, cystic degeneration of the lymph nodes was not associated with recurrence.

The κ values for the two observers revealed fair agreement regarding the margin of the primary lesion and marginal invasion (0.31–0.38); moderate agreement regarding necrosis, marginal invasion of the primary lesion, and ENE (0.48–0.50); and substantial agreement regarding cystic nodal metastasis and the unenhanced area of nodal metastasis (0.64–0.79).

## 4. Discussion

In this study, the recurrence rate was lower in HPV-positive OPSCC than in HPV-negative OPSCC. The T and N categories of HPV-negative OPSCCs were associated with recurrence, whereas those of HPV-positive OPSCCs did not differ between patients with and without recurrence. Regarding nodal metastases in HPV-negative OPSCC, attenuation on contrast-enhanced CT images and SIR on contrast-enhanced T1-weighted images were significantly lower in patients with recurrence than in those without. In HPV-positive and HPV-negative OPSCCs, cystic changes did not differ significantly between patients with and without recurrence.

In this study, the recurrence rate of HPV-positive OPSCC was lower than that of HPV-negative OPSCC. Many previous studies [15,16,17] have reported that HPV-positive OPSCC had a better prognosis when compared with HPV-negative OPSCC. Because of differing prognoses, the present eighth edition of the staging system establishes separate TNM classifications for HPV-positive and HPV-negative OPSCC.

This study observed no significant differences in TNM classification between HPV-positive OPSCC patients with and without recurrence. Meanwhile, HPV-negative OPSCC patients with and without recurrence showed significant differences in the T and N categories and the maximum diameter of the primary lesion. A previous study that assessed the prognosis of HPV-positive OPSCC using the seventh edition of the TNM classification system reported that TNM classification was not a prognostic factor [18]. Another study on OPSCC, which included 67 patients, 55 of whom were p16-positive, reported that the T category, according to the eighth edition of the TNM classification, was not a prognostic factor [19]. Chang et al. reported that lymph node metastasis in HPV-positive OPSCC patients had no significant effect on recurrence or mortality [20]. Conversely, several studies on HPV-negative OPSCC have reported that higher stages had a worse prognosis [20,21,22,23]. Although the prognosis of HPV-positive OPSCC patients may be unrelated to TNM classification, TNM classification is a critical prognostic factor for HPV-negative OPSCC patients.

The present study demonstrated that attenuation on contrast-enhanced CT images and SIR on contrast-enhanced T1-weighted images of nodal metastases were significantly lower in HPV-negative OPSCC patients with recurrence than in those without. Low attenuation and SIR on contrast-enhanced images may be caused by the histological heterogeneity of the keratinizing phenotype that is common in HPV-negative OPSCC [24]. Furthermore, ADC values for nodal metastases were lower in HPV-negative OPSCC patients with recurrence than in those without. Several studies analyzing OPSCC MRI results have reported that low ADC values were associated with HPV positivity and better prognosis [19,25]. However, among HPV-positive OPSCC patients, ADC values were not associated with overall survival [19]. Although no studies have reported a strong correlation between ADC values and prognosis in HPV-negative OPSCC patients, low ADC values are typically associated with high grade and poor prognosis. Low contrast enhancement and ADC values may predict the recurrence of HPV-negative OPSCC.

This study observed no significant difference in cystic change or necrosis between HPV-positive and HPV-negative patients with and without recurrence. Previous studies have reported that cystic nodal metastasis increases the risk of regional failure in OPSCC [26] and distant metastatic recurrence of nasopharyngeal carcinoma [27]. Another study concluded that cystic nodal metastasis indicated a low risk of recurrence compared with solid nodal metastasis in HPV-positive OPSCC [28]. Although cystic nodal metastasis helps in distinguishing between HPV-positive and HPV-negative OPSCC and suggests that HPV-positive OPSCC has a good prognosis, further study is required to determine the association between cystic nodal metastasis and prognosis.

In this study, ENE of nodal metastasis was associated with recurrence in HPV-negative OPSCC but not with recurrence in HPV-positive OPSCC. ENE was an independent predictor of survival in HPV-negative OPSCC patients [23]; thus, the eighth edition of the TNM classification system for OPSCC reflected ENE in only HPV-negative OPSCC. However, a recent meta-analysis reported that ENE in HPV-positive OPSCC patients is moderately associated with an increased risk of all-cause mortality and distant metastasis but not with locoregional recurrence [29]. The association between ENE and prognosis in HPV-positive OPSCC patients remains debatable.

This study has several limitations. First, the use of a single-center study resulted in a relatively small number of cases, especially among those with HPV-negative OPSCC and female patients. Gender distribution or unevenness in the number of HPV-positive and HPV-negative patients can affect the outcome. Second, CT and MRI images were obtained using various scanners. Finally, a survival analysis using the Kaplan–Meier curve was impossible because of the small number of cases.

## 5. Conclusions

In HPV-positive OPSCC, the T and N categories were not associated with recurrence, and no helpful imaging findings for predicting recurrence were found. However, T and N categories were associated with recurrence in HPV-negative OPSCC. In HPV-negative OPSCC, patients with recurrence had lower attenuation on contrast-enhanced CT images and SIR on contrast-enhanced T1-weighted images of nodal metastases than those without recurrence. Cystic lymph node metastasis was not associated with recurrence; however, ENE in HPV-negative OPSCC was associated with recurrence. Thus, prognostic factors differed considerably between HPV-positive and HPV-negative OPSCC.

## Figures and Tables

**Table 1:** Clinical characteristics of patients with OPSCC.

	All Patients			HPV-Positive OPSCC			HPV-Negative OPSCC		
	Recurrence		*p*	Recurrence		*p*	Recurrence		*p*
	Yes (*n* = 17)	No (*n* = 51)		Yes (*n* = 7)	No (*n* = 41)		Yes (*n* = 10)	No (*n* = 10)	
Age (years)	71 [67–75]	66 [52–71]	0.013	73 [70–75]	60 [51–68]	0.045	69 [67–74]	70 [52–76]	0.73
Male	7 (41%)	38 (75%)	>0.99	5 (71%)	30 (73%)	>0.99	8 (80%)	8 (80%)	>0.99
Smoking	13 (76%)	34 (67%)	0.55	5 (71%)	27 (66%)	>0.99	8 (80%)	7 (70%)	>0.99
Surgery	8 (47%)	37 (73%)	0.08	4 (57%)	12 (29%)	0.20	5 (50%)	8 (80%)	0.35
Chemotherapy	14 (82%)	28 (55%)	**0.049**	5 (71%)	23 (56%)	0.68	9 (90%)	5 (50%)	0.14
Radiation	15 (88%)	35 (69%)	0.20	6 (86%)	29 (70%)	0.66	9 (90%)	6 (60%)	0.30
T stage			**0.02**			0.52			**0.04**
T1	1 (6%)	9 (18%)		1 (14%)	6 (15%)		0 (0%)	3 (30%)	
T2	6 (35%)	31 (61%)		4 (57%)	26 (63%)		2 (20%)	5 (50%)	
T3	4 (24%)	2 (4%)		1 (14%)	1 (2%)		3 (30%)	1 (10%)	
T4	6 (35%)	9 (18%)		1 (14%)	8 (20%)		5 (50%)	1 (10%)	
N stage			0.35			>0.99			**0.04**
N0	1 (6%)	10 (20%)		1 (14%)	6 (15%)		0 (0%)	4 (40%)	
N1	8 (47%)	27 (53%)		5 (71%)	27 (66%)		3 (30%)	0 (0%)	
N2	7 (41%)	12 (24%)		1 (14%)	8 (20%)		6 (60%)	4 (40%)	
N3	1 (6%)	2 (4%)		0 (0%)	0 (0%)		1 (10%)	2 (20%)	
M stage			NA			NA			NA
M1	0 (0%)	0 (0%)		0 (0%)	0 (0%)		0 (0%)	0 (0%)	

Note.: OPSCC = oropharyngeal squamous cell carcinoma, HPV = human papilloma virus. Fisher’s exact test and Mann–Whitney U test were used. Qualitative data are numbers of patients with percentages in parentheses. Quantitative data are expressed as medians with interquartile range in square brackets. TNM classification was based on the eighth edition of staging system.

**Table 2:** Quantitative and qualitative imaging findings of primary lesion.

Quantitative Imaging Findings of Primary Lesion							
		HPV-Positive OPSCC			HPV-Negative OPSCC		
		Recurrence		*p*	Recurrence		*p*
		Yes (*n* = 7)	No (*n* = 41)		Yes (*n* = 10)	No (*n* = 10)	
Maximum diameter (mm)		21.9 [18.2–26.6]	28.4 [19.7–34.7]	0.18	32.7 [26.0–45.4]	20.8 [19.3–33.9]	**0.04**
***CT***							
Attenuation on UECT (HU)		50.3 [41.6–55.7]	50.4 [43.4–55.4]	0.81	42.1 [38.7–44.6]	49.4 [46.6–50.6]	**<0.01**
Attenuation on CECT (HU)		85.7 [82.3–93.7]	85.0 [78.4–97.3]	0.88	90.2 [76.1–95.2]	90.7 [83.8–97.8]	0.87
***MRI***							
SIR on T1WI		1.04 [0.98–1.06]	0.98 [0.93–1.06]	0.42	0.99 [0.92–1.03]	0.94 [0.92–0.99]	0.63
SIR on T2WI		1.12 [1.09–1.14]	1.06 [1.00–1.15]	0.37	1.02 [0.87–1.09]	1.16 [1.06–1.22]	0.089
SIR on DWI		1.66 [1.57–1.79]	1.32 [1.08–1.46]	<0.01	1.15 [0.99–1.36]	1.23 [0.95–1.55]	0.58
SIR on CET1WI		2.05 [1.92–2.21]	1.82 [1.63–1.99]	**0.02**	2.04 [1.76–2.19]	1.97 [1.91–2.07]	0.91
ADC value (×10^−3^ mm^2^/s)		0.82 [0.69–1.05]	0.80 [0.73–0.89]	0.98	0.91 [0.76–1.06]	1.02 [0.91–1.08]	0.51
***^18^F-FDG-PET/CT***							
SUVmax		15.3 [10.6–18.3]	12.61 [8.9–15.9]	0.58	16.5 [14.5–20.5]	18.5 [12.0–20.7]	0.96
**Qualitative imaging findings of primary lesion**							
***CT***							
Well-defined margin		5 (71%)	23 (56%)	0.68	5 (50%)	4 (40%)	>0.99
Necrosis		1 (14%)	9 (22%)	>0.99	2 (20%)	1 (10%)	>0.99
***MRI***							
Well-defined margin		7 (100%)	33 (80%)	0.58	4 (40%)	7 (70%)	0.37
Necrosis		2 (29%)	8 (20%)	0.63	3 (30%)	2 (20%)	>0.99
Marginal invasion		1 (14%)	6 (15%)	>0.99	5 (50%)	3 (30%)	0.65
T1WI	Hyperintensity Isointensity Hypointensity	1 (14%) 2 (29%) 4 (57%)	2 (5%) 19 (46%) 20 (49%)	0.41	1 (10%) 3 (30%) 6 (60%)	0 (0%) 5 (50%) 5 (50%)	0.65
T2WI	Hyperintensity Isointensity Hypointensity	5 (71%) 2 (29%) 0 (0%)	32 (78%) 8 (20%) 1 (2%)	0.68	7 (70%) 2 (20%) 1 (10%)	9 (90%) 0 (0%) 1 (10%)	0.72
CET1WI	Hyperintensity	7 (100%)	41 (100%)	NA	10 (100%)	10 (100%)	NA
DWI	Hyperintensity Isointensity Hypointensity	6 (86%) 1 (14%) 0 (0%)	33 (80%) 5 (12%) 3 (7%)	>0.99	10 (100%) 0 (0%) 0 (0%)	8 (80%) 1 (10%) 1 (10%)	0.47

Note: HPV = human papilloma virus, OPSCC = oropharyngeal squamous cell carcinoma, UECT = unenhanced CT, CECT = contrast-enhanced CT, SIR = signal intensity ratio, T1WI = T1-weighted images, T2WI = T2-weighted images, DWI = diffusion-weighted images, CET1WI = contrast-enhanced T1-weighted images, ADC = apparent diffusion coefficient, FDG = fluorodeoxyglucose, SUV = standardized uptake value. Fisher’s exact test and Mann–Whitney U test were used. Qualitative data are numbers of patients with percentages in parentheses. Quantitative data are expressed as medians with interquartile ranges in square brackets.

**Table 3:** Quantitative imaging findings of cervical lymph node metastasis.

	Nodal Metastasis From HPV-Positive OPSCC			Nodal Metastasis From HPV-Negative OPSCC		
	Recurrence		*p*	Recurrence		*p*
	Yes (*n* = 24)	No (*n* = 96)		Yes (*n* = 24)	No (*n* = 6)	
Maximum diameter (mm)	17.8 [14.0–22.5]	16.8 [13.5–22.0]	0.15	18.5 [15.3–26.5]	19.3 [16.5–22.3]	0.83
***CT (HU)***						
UECT	44.1 [38.6–47.9]	47.1 [44.4–51.6]	**0.01**	45.6 [42.8–48.8]	41.2 [34.3–47.1]	0.27
CECT	82.2 [71.5–96.7]	86.2 [75.4–98.4]	0.53	79.4 [68.2–84.3]	87.9 [86.8–95.6]	**0.03**
Necrosis of NCCT	38.3 [31.4–41.0]	33.6 [27.2–44.2]	0.45	31.4 [25.8–40.2]	28.3 [27.5–34.0]	0.82
Necrosis of CECT	49.0 [36.8–66.5] (*n* = 13)	42.3 [33.2–55.5] (*n* = 62)	0.28	39.3 [29.8–47.0] (*n* = 17)	33.9 [31.4–56.6] (*n* = 5)	0.65
***MRI***						
SIR on T1WI	1.00 [0.93–1.04]	0.97 [0.90–1.02]	0.14	0.94 [0.89–1.02]	0.89 [0.87–0.93]	0.35
SIR on T2WI	1.09 [0.96–1.36]	1.12 [0.99–1.32]	0.69	1.23 [0.95–1.37]	1.31 [1.15–1.47]	0.16
SIR on DWI	1.43 [1.12–1.62]	1.47 [1.19–1.83]	0.67	1.49 [1.18–1.72]	1.50 [1.45–2.11]	0.44
SIR on CE-T1WI	1.90 [1.74–2.04]	1.79 [1.57–2.03]	0.17	1.79 [1.64–2.08]	2.41 [1.97–2.38]	**0.04**
ADC value (×10^−3^ mm^2^/s)	0.87 [0.68–0.99]	0.86 [0.76–1.05]	0.34	0.83 [0.72–1.02]	1.13 [0.99–1.37]	0.06
***^18^F-FDG-PET/CT***						
SUVmax	9.52 [5.91–12.7]	7.78 [4.51–11.0]	0.28	7.86 [4.40–15.5]	4.31 [3.78–4.78]	0.07

Note: HPV = human papilloma virus, OPSCC = oropharyngeal squamous cell carcinoma, UECT = unenhanced CT, CECT = contrast-enhanced CT, SIR = signal intensity ratio, T1WI = T1-weighted images, T2WI = T2-weighted images, DWI = diffusion-weighted images, CE-T1WI = contrast-enhanced T1-weighted images, ADC = apparent diffusion coefficient, FDG = fluorodeoxyglucose, SUV = standardized uptake value. Fisher’s exact test and Mann–Whitney U test were used. Quantitative data are expressed as medians with interquartile ranges in square brackets.

**Table 4:** Qualitative imaging findings of cervical lymph node metastasis.

		Nodal Metastasis From HPV-Positive OPSCC			Nodal Metastasis From HPV-Negative OPSCC		
		Recurrence		*p*	Recurrence		*p*
		Yes (*n* = 24)	No (*n* = 96)		Yes (*n* = 24)	No (*n* = 9)	
***CT***							
ENE		2 (8%)	8 (8%)	>0.99	1 (4%)	4 (44%)	**0.01**
Unenhanced area - pure cystic - pure cystic + mural nodule - necrosis		12 (50%) 4 (17%) 1 (4%) 7 (29%)	62 (65%) 7 (7%) 14 (15%) 41 (43%)	0.24 0.15	17 (71%) 3 (13%) 4 (17%) 10 (42%)	6 (67%) 2 (22%) 0 (0%) 4 (44%)	>0.99 0.55
***MRI***							
ENE		2 (8%)	8 (8%)	>0.99	1 (4%)	4 (44%)	**0.01**
Unenhanced area - pure cystic - pure cystic + mural nodule - necrosis		18 (75%) 5 (21%) 3 (13%) 10 (42%)	64 (67%) 5 (5%) 14 (15%) 46 (48%)	0.48 0.08	18 (75%) 4 (17%) 3 (13%) 10 (42%)	8 (89%) 1 (11%) 1 (11%) 6 (67%)	0.64 0.72
Solid component							
T1WI	Hyperintensity Isointensity Hypointensity	0 (0%) 15 (63%) 9 (37%)	0 (0%) 40 (42%) 56 (58%)	0.11	3 (13%) 12 (50%) 9 (37%)	1 (11%) 2 (22%) 6 (67%)	0.35
T2WI	Hyperintensity Isointensity Hypointensity	12 (50%) 11 (46%) 1 (4%)	59 (61%) 30 (31%) 7 (7%)	0.50	16 (67%) 7 (29%) 1 (4%)	5 (56%) 3 (33%) 1 (11%)	0.70
DWI	Hyperintensity Isointensity Hypointensity	20 (83%) 4 (17%) 0 (0%)	85 (89%) 9 (9%) 2 (2%)	0.55	21 (88%) 2 (8%) 1 (4%)	7 (78%) 2 (22%) 0 (0%)	0.68
CE-T1WI	Hyperintensity Isointensity Hypointensity	24 (100%) 0 (0%) 0 (0%)	95 (99%) 1 (1%) 0 (0%)	>0.99	24 (100%) 0 (0%) 0 (0%)	9 (100%) 0 (0%) 0 (0%)	NA

Note: HPV = human papilloma virus, OPSCC = oropharyngeal squamous cell carcinoma, ENE = extranodal extension, T1WI = T1-weighted images, T2WI = T2-weighted images, DWI = diffusion-weighted images, CE-T1WI = contrast-enhanced T1-weighted images. Fisher’s exact test and Mann–Whitney U test were used. Qualitative data are numbers of patients with percentages in parentheses.

## Data Availability

The data presented in this study may be available on request from the corresponding author. The data are not publicly available due to data patient privacy concerns.
